# Association of Insurance Changes With Postpartum Prescription Contraception Uptake

**DOI:** 10.1097/og9.0000000000000047

**Published:** 2024-11-21

**Authors:** Kimberly M. Schaefer, Michele R. Hacker, Summer S. Hawkins, Rose L. Molina

**Affiliations:** Harvard Medical School, the Department of Obstetrics and Gynecology, Beth Israel Deaconess Medical Center, the Department of Obstetrics, Gynecology and Reproductive Biology, Harvard Medical School, and the School of Social Work, Boston College, Boston, Massachusetts; and the Department of Obstetrics & Gynecology, Oregon Health & Science University, Portland, Oregon.

## Abstract

**OBJECTIVE:**

To assess the association between postpartum insurance churn and postpartum contraception, with specific attention to race, ethnicity, language, and Medicaid expansion status.

**METHODS:**

We conducted a retrospective cohort study with Pregnancy Risk Assessment Monitoring System survey data (2012–2020) from 42 states. Insurance status, assessed more than 2 months after childbirth, was categorized as continuous insurance, insurance loss, discontinuous Medicaid-to-private, discontinuous private-to-Medicaid, continuous uninsurance, and gain of insurance. We used logistic regression to estimate odds ratios with 95% CIs for insurance status and use of prescription contraception methods, compared with nonprescription and no method, and adjusted for age, race and ethnicity, language, marital status, percent of federal poverty level, education, and birth year. We considered the interaction between state Medicaid expansion and insurance status and stratified analyses by race, ethnicity, and language.

**RESULTS:**

Of 223,430 respondents, 78.9% held continuous insurance from childbirth to more than 2 months postpartum and 10.2% lost insurance. Compared with continuous insurance, the adjusted odds ratio (95% CI) for prescription postpartum contraception was 0.74 (0.71–0.78) for insurance loss, 0.90 (0.84–0.97) for discontinuous private-to-Medicaid, and 1.04 (0.98–1.10) for discontinuous Medicaid-to-private. There was no evidence for an interaction between living in states with Medicaid expansion and insurance status. Of respondents without Medicaid expansion, 14.0% (95% CI, 13.6–14.3) experienced insurance loss compared with 6.8% (95% CI, 6.6–7.0) with Medicaid expansion. There was a significant interaction between insurance discontinuity and race, ethnicity, and language. Nearly half of the Hispanic Spanish-language respondents (47.5%) experienced insurance loss postpartum.

**CONCLUSION:**

Insurance loss more than 2 months postpartum was associated with decreased odds of prescription contraception. Populations that experience higher rates of insurance loss include respondents in states without Medicaid expansion and Hispanic Spanish-language respondents. Medicaid and insurance policies have important roles in enabling coverage for contraceptive access across racial and ethnic groups during the postpartum period.

## Abstract 2

Postpartum insurance loss is associated with decreased likelihood of prescription contraception, with higher insurance loss rates among Hispanic Spanish-language respondents and those without Medicaid expansion.

Changes in insurance coverage, known as insurance churn, have been associated with fragmentation of care and adverse health outcomes, including disruptions in medication adherence, increased emergency department use, and worse self-reported health status.^1–3^ The postpartum period, including up to 1 year after childbirth,^4^ is especially vulnerable to this churn. The higher income thresholds for pregnancy-related Medicaid coverage typically lapse 60 days after birth; studies have shown that more than one-fifth of birthing people with pregnancy-related Medicaid coverage become uninsured after this period.^5,6^ There are both state and federal efforts to extend this pregnancy-related coverage to 1 year, including Medicaid 1115 waivers and state plan amendments. The American Rescue Plan Act of 2021 introduced state plan amendments as a pathway for states to receive federal matching for postpartum coverage up to 1 year.^7^ As of December 14, 2023, 42 states have implemented 12-month postpartum extensions.^8^

Insurance status is also known to reflect racial and ethnic inequities in pregnancy care; across the prenatal, childbirth, and postpartum time periods, rates of uninsurance remain higher among Black, Indigenous, Hispanic, Asian/Pacific Islander women compared with White non-Hispanic women.^9^ Because uninsurance is linked to health access and outcomes, postpartum insurance coverage has emerged as an opportunity for policy to improve these inequities, especially during the late postpartum period wherein significant postpartum morbidity and mortality occurs.^4,10^

Postpartum contraception is an important part of pregnancy-related care, with a critical role in preventing unintended pregnancies and fostering reproductive autonomy.^11^ This study assesses the relationship between postpartum insurance churn and use of prescription contraceptive methods, with further attention to differences based on race, ethnicity, and language. Although prescription contraceptive methods are not the optimal choice for every postpartum individual, there is no clinical basis for insurance status alone to influence use patterns. However, given that insurance can be a considerable barrier to care, we hypothesized that discontinuous insurance coverage from pregnancy to more than 2 months after birth would be associated with lower rates of prescription contraceptive method use.

## METHODS

We conducted a retrospective cohort study using Pregnancy Risk Assessment Monitoring System (PRAMS) survey data from 2012 to 2020 in 42 states. This survey, administered by the Centers for Disease Control and Prevention’s (CDC) Division of Reproductive Health in collaboration with state health departments, collects individual experiences before, during, and after pregnancy.^12^ Surveys are administered by mail and telephone. Sampling is stratified on demographic indicators to be representative of the pregnant population and data are linked to birth certificates for demographic and medical information; full survey methodology is published by the CDC.^13^

We limited our sample to respondents who completed the survey more than 2 months after birth, based on the minimum interval of pregnancy-related Medicaid coverage, and up until 1 year postpartum. This interval was calculated using date of survey completion and infant date of birth, as recorded by birth certificate data. Respondents were removed from analysis if either of these two dates were missing and they, therefore, had an unknown interval from birth. We also limited the sample to respondents with complete information on insurance coverage and postpartum contraception use. We further excluded respondents who were missing data for any of the prespecified covariates for a complete case analysis and assessed demographics of the missing data.

The primary exposure was insurance discontinuity, and the primary outcome was postpartum prescription contraception, with prespecified covariates of age, race and ethnicity, language, state, marital status, percent of federal poverty level (FPL), education, and birth year. We identified postpartum visit attendance as an effect modifier and, thus, did not include it in our primary model. Insurance coverage was assessed at preconception (reported as 1 month before pregnancy), birth, and postpartum time periods. For each period, insurance was categorized as Medicaid, private, and uninsured. “Other” and “uninsured” were both reportable answers in the surveys; free-text responses were available for the “other” category, and this group was reclassified accordingly. “Medicaid” included respondents who answered Medicaid, Children's Health Insurance Program, or state government insurance. “Private” included respondents who answered insurance from work, parents, military, TRICARE, or the insurance marketplace. For insurance coverage at childbirth, data are available from birth certificate payor information and recorded within PRAMS. We used this as the primary data source and filled in the remaining missing insurance data from self-reported survey responses as available. We generated six categories that compared insurance status at birth to postpartum periods: continuous insured, discontinuous Medicaid-to-private, discontinuous private-to-Medicaid, discontinuous loss of insurance, continuous uninsured, and gain of insurance (Appendix 1, available online at http://links.lww.com/AOG/D912).

Income is reported in categorical increments; however, the income cutoff values vary by individual state surveys. We used the median values of each income band as an estimate and converted this value to percent of FPL using the respondent's reported number of dependents in relation to FPL thresholds based on year and state by the U.S. Department of Health and Human Services.^14^

Survey respondents reported yes or no to each method of contraception included on the survey (abstinence, withdrawal, rhythm, condoms, oral contraceptive pill, patch or ring, contraceptive injection, intrauterine device [IUD], implant, tubal ligation or salpingectomy, and vasectomy). We categorized these hierarchically by effectiveness using CDC classifications of effectiveness.^15^ If participants responded “yes” to more than one method, the use of a more effective method superseded that of a less effective one. For respondents who were missing a response to all-of-the-above methods but had filled out free text answers, we reclassified their responses into the appropriate category based on the free text. We further classified contraceptive method into a dichotomous indicator of none or least effective and moderately or most effective. By CDC classifications, none or least effective included no method, abstinence, breastfeeding, withdrawal, rhythm, condom, and gel or spermicide; moderately or most effective included patch or ring, pill, injection, diaphragm, IUD, implant, tubal ligation or salpingectomy, and vasectomy.^15^ We refer to these as “nonprescription” and “prescription” methods, respectively, to place them in the context of insurance coverage. We removed respondents who reported same sex, infertility, hysterectomy, and current pregnancy as free text responses.

To account for differential enactment of Medicaid expansion over this time period, we coded all states as “nonexpansion” before 2014 (beginning of Medicaid expansion), as “expansion” after 2014 if they were part of the initial Medicaid expansion, and as “nonexpansion” if they had not expanded Medicaid as of 2020. For the remaining states that enacted Medicaid expansion at some point between 2014 and 2020, we identified the month and year of Medicaid expansion in each state^16^ and categorized respondents accordingly based on month and year of the infant's date of birth.

Race and ethnicity information in the PRAMS survey data is extracted from birth certificates, on which it is self-reported. The options specified in the PRAMS data are Asian, White, Black, American Indian, Chinese, Japanese, Filipino, Hawaiian, Alaskan Native, mixed race, other Asian, other non-White, and unknown. Hispanic (yes or no) was a separate birth certificate item. We consolidated these into six distinct groups: White non-Hispanic, Black non-Hispanic, Asian American and Pacific Islander (including Chinese, Japanese, Filipino, Hawaiian, other Asian), Indigenous (American Indian and Alaskan Native), mixed/unknown/other (mixed race, other non-White, and missing data), and Hispanic. Data were missing for 7,249 respondents (3.2%) who were included in the mixed/unknown/other group. Given the established disparities in perinatal outcomes related to immigration status among Hispanic patients,^9,17,18^ we created an additional categorization by race, ethnicity, and language, based on the language that the survey was collected in (Hispanic English-language and Hispanic Spanish-language) to further assess possible immigration and language-related barriers to care.

We calculated the unadjusted, weighted proportion of respondents in each of the six insurance discontinuity groups (continuous insured, discontinuous private-to-Medicaid, discontinuous Medicaid-to-private, uninsured, loss of insurance, and gain of insurance), and descriptive statistics for each of the insurance groups. Our primary analysis used multivariable logistic regression to estimate the odds ratio (OR) for the association between insurance discontinuity and prescription contraception use, adjusted for all prespecified covariates. For the exposure, we used the six insurance discontinuity groups previously described, and we defined the outcome as dichotomous (prescription vs nonprescription contraceptive method).

To assess differences in outcomes by Medicaid expansion status, we included state Medicaid expansion as an interaction term with insurance status and used an adjusted Wald test to assess significance of the interaction. We repeated these steps for a single variable capturing race, ethnicity, and language as an interaction term with insurance status. Because both interaction terms had several levels, if the interaction was significant by the Wald test (*P*<.05), we subsequently performed a stratified analysis to aid in interpretability.

We accounted for the complex survey design by applying Stata's survey command to the survey weights provided with the PRAMS data set.^13^ All analyses were conducted using Stata 16.1. PRAMS protocols are reviewed and approved by the CDC’s IRB and all local IRBs.^12^ This study is a secondary analysis of deidentified data that is not considered human subjects research and, therefore, did not require IRB review. We followed STROBE (Strengthening the Reporting of Observational Studies in Epidemiology) reporting guidelines for observational studies.

## RESULTS

Demographic characteristics of the respondents are shown in Table 1. This represented 83.2% of the initial data set (Appendix 2, available online at http://links.lww.com/AOG/D912); the bulk of missing data was from income or the number of dependents, and, thus, FPL (13.8%; see Appendices 3–5, available online at http://links.lww.com/AOG/D912). For complete case analysis, 3.5% of the respondents were excluded because of missing information on at least one of the covariates. Of the 223,430 respondents included in the final analysis, 78.9% maintained continuous coverage from childbirth to the 2^nd^ through the 12^th^ month postpartum period, and 21.1% experienced some form of insurance discontinuity. Overall, respondents with continuous insurance tended to be older, White, and married; have higher income and education levels; reside in a Medicaid-expansion state; and report English as a primary language.

**Table 1.** Population Demographics by Insurance Continuity Status at 2–12 Months Postpartum Among Pregnancy Risk Assessment Monitoring System Respondents, 2012–2020*

Characteristic	Continuous Insured [n=173,267 (78.9)]	Discontinuous		Uninsured [n=1,134 (0.26)]	Loss of Insurance [n=25,152 (10.2)]	Gain of Insurance [n=629 (0.11)]	Overall (N=223,430)
		Private to Medicaid [n=8,878 (4.00)]	Medicaid to Private [n=14,370 (6.51)]				
Age (y)							
19 or younger	7,005 (3.4)	699 (6.5)	1,176 (7.80)	57 (3.5)	1,465 (5.1)	60 (7.61)	10,462 (4.0)
20–24	28,980 (16.0)	2,279 (27.0)	4,469 (31.8)	251 (22.0)	6,621 (26.4)	154 (20.7)	42,754 (18.6)
25–29	50,545 (29.5)	2,780 (33.1)	4,157 (30.0)	388 (31.0)	7,932 (32.1)	186 (37.4)	65,897 (29.9)
30–34	54,454 (31.2)	1,947 (20.9)	2,835 (19.1)	282 (27.4)	5,715 (22.7)	136 (22.6)	65,370 (29.9)
35 or older	32,374 (18.9)	1,172 (12.7)	1,733 (11.3)	156 (16.2)	3,419 (13.7)	93 (11.6)	38,947 (17.6)
Education							
Less than high school	14,989 (7.5)	1,293 (14.6)	2,131 (15.3)	317 (40.7)	6,684 (27.8)	143 (22.1)	25,557 (10.5)
High school	35,998 (20.1)	2,891 (33.9)	47,171 (33.1)	379 (30.5)	8,736 (34.6)	208 (37.9)	52,929 (23.0)
Some college	49,744 (27.4)	3,405 (38.2)	5,261 (36.1)	305 (16.0)	7,275 (26.9)	178 (21.7)	66,168 (28.3)
College or more	72,536 (45.0)	1,289 (13.3)	2,261 (15.6)	133 (12.8)	2,457 (10.8)	100 (18.3)	78,776 (38.2)
Race and ethnicity							
Asian	14,619 (6.2)	486 (3.5)	834 (5.2)	30 (4.3)	923 (3.20)	288 (3.9)	16,920 (5.7)
Black	26,902 (12.2)	2,406 (21.5)	3,629 (21.3)	39 (5.7)	3,094 (11.4)	33 (8.0)	36,103 (13.0)
Hispanic	18,231 (9.9)	1,566 (17.1)	2,511 (17.3)	347 (47.6)	8,439 (36.3)	91 (24.1)	31,185 (13.5)
Indigenous	13,879 (3.5)	701 (4.4)	1,070 (4.4)	166 (6.2)	1,657 (4.1)	67 (10.1)	17,540 (3.7)
Mixed/unknown/other	4,012 (0.6)	194 (0.50)	558 (1.0)	408 (12.6)	2,474 (2.6)	279 (18.8)	7,925 (0.9)
White	95,624 (67.6)	3,525 (53.1)	5,768 (50.8)	144 (23.7)	8,565 (42.3)	131 (35.1)	113,757 (63.2)
% FPL							
Less than 138	57,926 (29.7)	5,584 (64.0)	7,994 (54.6)	677 (68.8)	17,690 (68.4)	360 (58.9)	90,150 (36.8)
138–199	17,343 (9.6)	1,509 (17.4)	2,676 (18.3)	132 (14.4)	3,687 (15.4)	71 (10.6)	25,418 (11.1)
200–399	61,833 (38.9)	1,600 (16.6)	3,164 (23.1)	237 (14.0)	3,466 (14.7)	149 (26.1)	70,449 (34.5)
400 or more	36,165 (21.7)	185 (2.0)	536 (4.0)	88 (2.8)	390 (1.6)	49 (4.4)	37,413 (17.6)
Marital status							
Not married	57,974 (30.8)	5,513 (63.8)	7,910 (55.6)	564 (50.4)	13,310 (51.1)	342 (46.7)	85,613 (35.9)
Married	115,293 (69.2)	3,365 (36.2)	6,460 (44.4)	570 (49.6)	11,842 (48.9)	287 (53.3)	137,817 (64.1)
Language							
English	168,131 (96.9)	8,371 (94.2)	13,258 (91.8)	879 (63.2)	19,283 (73.8)	582 (86.1)	210,504 (94.0)
Spanish	4,806 (2.7)	502 (5.6)	1,070 (7.7)	255 (36.8)	5,835 (26.0)	47 (13.9)	12,515 (5.7)
Chinese	330 (0.4)	5 (0.1)	42 (0.5)	0 (0)	34 (0.3)	0 (0)	411 (0.3)

FPL, federal poverty level.

Data are unweighted sample size (weighted proportion).

* The weighted proportion was determined using Stata's survey command, with the survey weights provided with the Pregnancy Risk Assessment Monitoring System data set; these percentages may, therefore, be different from unweighted percentages.

Unadjusted and adjusted ORs for prescription contraceptive methods by insurance discontinuity status are presented in Table 2. In the adjusted model, loss of insurance was associated with 26% lower odds for prescription contraception compared with continuous insurance (OR 0.74, 95% CI, 0.71–0.78). Discontinuous private-to-Medicaid also demonstrated a significantly decreased, though smaller, odds for prescription contraception compared with continuous insurance (OR 0.90, 95% CI, 0.84–0.97). Continuous uninsurance and gain of insurance were both associated with decreased odds of prescription contraception. We did not observe an association between discontinuous Medicaid-to-private insurance and prescription contraception (OR 1.04, 95% CI, 0.98–1.10).

**Table 2.** Odds of Prescription Contraception by Insurance Discontinuity at 2–12 Months Postpartum Among Pregnancy Risk Assessment Monitoring System Respondents, 2012–2020 (N=223,430)

Insurance Status	Unadjusted OR (95% CI)	Adjusted OR (95% CI)*
Continuous insured	Ref	
Discontinuous		
Private to Medicaid	1.19 (1.10–1.27)	0.90 (0.84–0.97)
Medicaid to private	1.31 (1.24–1.38)	1.04 (0.98–1.10)
Loss of insurance	0.96 (0.92–1.00)	0.74 (0.71–0.78)
Uninsured	0.64 (0.51–0.82)	0.51 (0.40–0.64)
Gain of insurance	0.87 (0.64–1.19)	0.69 (0.51–0.93)

OR, odds ratio.

* Adjusted for age, race and ethnicity, language, marital status, percent of federal poverty level, education, and year.

**Fig. 1.**
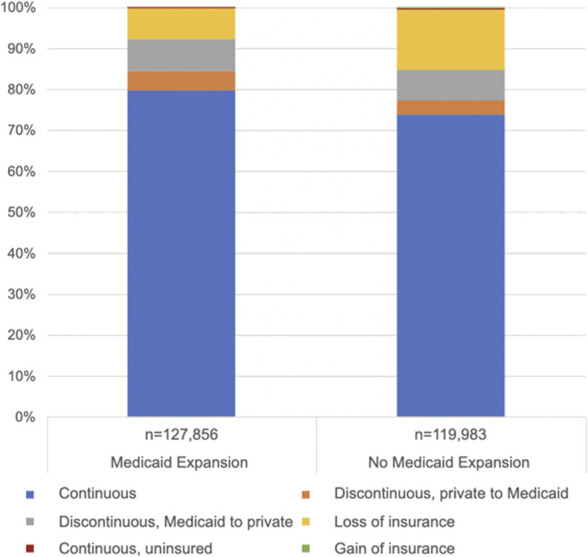
Distribution of insurance discontinuity by state Medicaid expansion status among Pregnancy Risk Assessment Monitoring System respondents, 2012–2020.

The distribution of insurance discontinuity by Medicaid expansion status is shown in Figure 1. Although a greater percentage of patients who were not in Medicaid-expansion states experienced insurance loss in the postpartum period (14.0%, 95% CI, 13.6–14.3) compared with Medicaid-expansion states (6.8%, 95% CI, 6.6–7.0), there was no evidence for a significant interaction between Medicaid expansion and insurance discontinuity (*P*=.15).

*Schaefer. Postpartum Insurance Churn and Contraception. O&G Open 2024*.

**Fig. 2.**
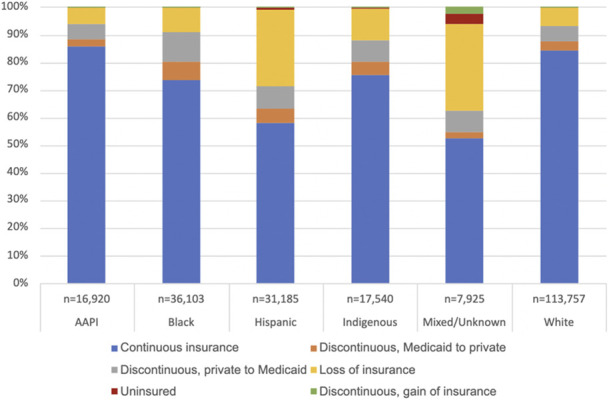
Distribution of insurance discontinuity status by race and ethnicity among Pregnancy Risk Assessment Monitoring System respondents, 2012–2020. AAPI, Asian American Pacific Islander. The Mixed/Unknown category includes participants who responded “mixed race” or “other non-White” and those with missing data.

**Fig. 3.**
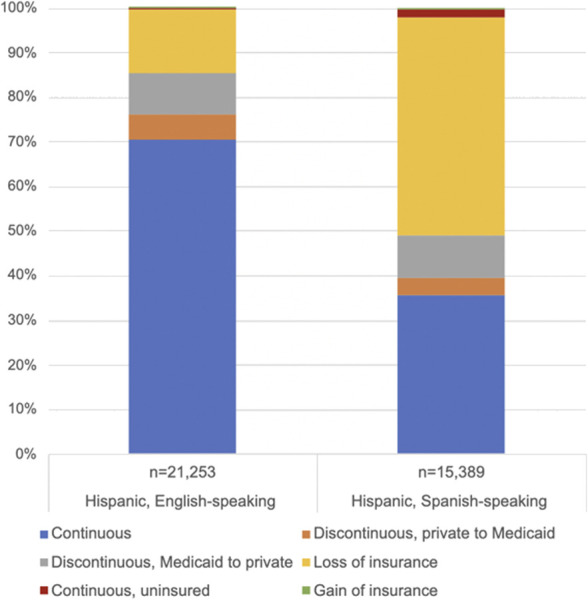
Distribution of insurance discontinuity by language status among Pregnancy Risk Assessment Monitoring System respondents, 2012–2020.

Figure 2 depicts the distribution of insurance discontinuity status by race, ethnicity, and language, with percentages as weighted sample proportions. Higher percentages of Hispanic and respondents of mixed or unknown racial backgrounds fell into the loss of insurance category (27.5% [95% CI, 26.8–28.3] and 31.4% [95% CI, 29.6–33.3], respectively). Within the Hispanic category, the Hispanic Spanish-language group had a larger percentage of loss of insurance (47.5% [95% CI, 46.2–48.9]) and smaller percentage of continuous insurance (37.4% [95% CI, 36.1–38.7]) compared with the Hispanic English-language group (14.0% [95% CI, 13.2–14.7] and 71.9% [95% CI, 70.9–72.9], respectively; Fig. 3). There was a significant interaction between the race-ethnicity-language variable and insurance discontinuity (*P*<.001). In the stratified analysis (Table 3), the association between discontinuous private-to-Medicaid was significant only in the White and Hispanic English-language populations; however, sample sizes for Asian American and Pacific Islander, Indigenous, and Hispanic Spanish-language groups were relatively small, which may limit interpretation of nonsignificant results. The association between discontinuous, Medicaid-to-private was not significant for any racial or ethnic groups. Loss of insurance was significantly associated with decreased odds of prescription contraception among Black and Hispanic English-language subgroups, the mixed/unknown/other subgroup, and White subgroups. Gain of insurance is associated with higher odds of prescription contraception in the Hispanic Spanish-language group.

*Schaefer. Postpartum Insurance Churn and Contraception. O&G Open 2024*.

*Schaefer. Postpartum Insurance Churn and Contraception. O&G Open 2024*.

**Table 3.** Odds of Prescription Postpartum Contraception by Insurance Discontinuity at 2–12 Months Postpartum Among Pregnancy Risk Assessment Monitoring System Respondents, 2012–2020, Stratified by Race, Ethnicity, and Language*

Insurance Status	AAPI	Black	Hispanic		Indigenous	Mixed/Unknown/Other	White
			English Language	Spanish Language			
Continuous insured	Ref	Ref	Ref	Ref	Ref	Ref	Ref
Discontinuous							
Private to Medicaid	1.34 (0.99–1.82)	0.93 (0.80–1.08)	0.77 (0.64–0.93)	0.93 (0.71–1.21)	0.72 (0.40–1.32)	0.78 (0.58–1.06)	0.90 (0.82–1.00)
Medicaid to private	1.00 (0.79–1.25)	1.10 (0.98–1.25)	0.98 (0.82–1.16)	1.09 (0.88–1.36)	1.04 (0.75–1.46)	1.08 (0.84–1.40)	1.03 (0.95–1.12)
Loss of insurance	0.96 (0.76–1.21)	0.77 (0.68–0.89)	0.76 (0.67–0.87)	0.94 (0.84–1.07)	0.89 (0.74–1.08)	0.65 (0.53–0.80)	0.68 (0.63–0.72)
Uninsured	0.27 (0.08–0.90)	0.48 (0.19–1.22)	0.89 (0.43–1.87)	0.68 (0.43–1.08)	0.86 (0.63–1.17)	0.50 (0.30–0.86)	0.26 (0.15–0.43)
Gain of insurance	0.79 (0.29–2.18)	0.84 (0.27–2.64)	0.51 (0.19–1.39)	2.94 (1.15–7.51)	1.27 (0.91–1.80)	0.38 (0.17–0.87)	0.40 (0.23–0.67)

AAPI, Asian American and Pacific Islander; Ref, reference.

Data are adjusted odds ratio (95% CI)

* Adjusted for age, marital status, percent of federal poverty level, education, and year.

## DISCUSSION

Using a large nationally representative data set, we found that changes in insurance 2 months after childbirth were associated with decreased likelihood of using prescription contraception methods. Loss of insurance was associated with the lowest odds of postpartum prescription contraception. This finding is consistent with studies that have shown uninsured patients to have lower odds of long-acting reversible contraceptive methods than insured patients.^19^ Without insurance, these forms of contraception can be cost prohibitive; out-of-pocket costs for IUD devices, alone, can cost more than of $1,000, in addition to appointment and insertion fees.^20^ The other forms of insurance churn we analyzed are private insurance during childbirth to Medicaid insurance postpartum, and, conversely, Medicaid insurance at birth to private insurance postpartum. We see no association of prescription contraception in the Medicaid-to-private group; however, we do see significantly decreased odds of prescription contraception in the private-to-Medicaid group. Although these both represent insurance discontinuity, it may be that other circumstances that beget a gain compared with loss of private insurance, such as job loss, may also influence access to postpartum care and that individuals who change to Medicaid insurance postpartum may face increased barriers to establishing care, because fewer practices accept Medicaid insurance.^21^

Our analysis stratified by race and ethnicity showed mixed results between insurance churn and postpartum contraception. We found that Hispanic respondents, particularly Spanish-language Hispanic respondents, and respondents of mixed or unknown racial backgrounds experienced loss of insurance postpartum. Similar to the primary analysis, loss of insurance was associated with lower odds of prescription contraception among Black and Hispanic English-language subgroups, the mixed/unknown/other subgroup, and White subgroups; the small sample sizes among Indigenous and Asian American and Pacific Islander strata, however, limit interpretability of these nonsignificant associations. This is taken in context of the pervasive racial inequities in pregnancy outcomes and the racism that belies them^22^; such disparities are evident in insurance coverage with a much larger proportion of Black and Hispanic birthing people covered by Medicaid (64% and 58% of births, respectively) compared with other racial and ethnic groups (ranging from 22 to 29%).^23^

Relatedly, Daw et al^9^ found that Black, Indigenous, and Hispanic Spanish-language patients had significantly lower rates of continuous insurance from preconception to postpartum. As a result, these racial and ethnic groups are disproportionately exposed to the deleterious effects of insurance churn, which may include, as our study suggests, a lower likelihood of prescription, and, therefore, more effective, postpartum contraception. In our analysis with ethnicity and language, we saw a large proportion of the Hispanic Spanish-language group with loss of insurance postpartum.

Given the intersection of language and immigration status,^24^ it is likely that a significant contributing factor is the use of Emergency Medicaid among immigrants in this group who do not qualify for full Medicaid (ie, undocumented individuals). Emergency Medicaid is subject to different regulations than traditional Medicaid, and, in the majority of states, this form of Medicaid covers only labor and delivery, with no postpartum care coverage.^25,26^ As a result, a greater proportion of these patients are also at risk for the health sequelae of insurance loss. In addition, although gain of insurance across all groups is rare given the overall high rates of insurance at time of delivery, gain of insurance in this population is associated with increased odds of prescription contraception, which further supports the importance of insurance coverage for this population. That a greater percent of minoritized groups is vulnerable to these effects of postpartum insurance churn remains an inequity that can be addressed, at least in part, by policy changes to Medicaid coverage.

Consistent with studies that examine the association of Medicaid expansion and postpartum contraception,^27,28^ we find significantly increased odds of more effective forms of contraception with lower rates of insurance churn. Medicaid expansion has been shown to be associated largely with improved perinatal health outcomes, including a 17% reduction in hospitalization during the first 60 days postpartum,^29^ decreased postpartum uninsurance and insurance churn,^5,6,30,31^ and decreased racial inequities in postpartum coverage.^32^ With regard to contraception, specifically, Medicaid expansion is associated with an increase in use of any postpartum contraception,^27^ especially more effective forms of contraception.^28^ Although state Medicaid expansion status was not a primary exposure in our analysis, various studies have shown that among people with Medicaid-covered births, those who live in nonexpansion states have higher rates of postpartum uninsurance,^5,6^ a result confirmed in our study (Fig. 1). Expansion status, however, did not have a significant interaction with the odds of postpartum contraceptive use and insurance churn; this is likely due to the further upstream effects of Medicaid expansion in affecting the rates of insurance churn itself rather than the sequelae of this insurance churn.

This study has several limitations. Although we used a large nationally representative data set with weighted sampling, limitations of the PRAMS data include the exclusion of data from several states (Arizona, California, Florida, Idaho, Indiana, Nevada, South Carolina, and Texas), which may limit the generalizability of our findings. We did not have the information to examine insurance discontinuity that took place within the same insurance strata (eg, Medicaid to Medicaid managed care organizations), which may contribute to an underestimate in the discontinuity population. Although we include language as a lens for analysis, we cannot specifically examine the use of Emergency Medicaid, as compared with full Medicaid, for individuals who may be undocumented, for whom more sweeping changes to Emergency Medicaid eligibility and coverage are needed for expanded access. This study also was limited by data completeness, specifically for income; although the results do not appear to change based on this (Appendices 3 and 4, http://links.lww.com/AOG/D912), it may nonetheless introduce sampling bias. Finally, because this was an unfunded study, we were unable to engage community members and patients in the research design and process for interpreting findings.

The increasingly widespread uptake of state plan amendments to extend postpartum Medicaid coverage from 60 days to 1 year with federal funding is a significant step toward improving continuity of postpartum insurance coverage.^8^ As the results of these policy changes unfold, future work will assess the effects of extended postpartum Medicaid coverage. For states that do not move in this direction, this study highlights the need for greater affordability of contraception to minimize cost barriers related to uninsurance. In this post–*Roe v. Wade* era with increasingly restrictive abortion bans, access to effective contraception becomes even more critical to uphold reproductive autonomy. This study suggests that with decreased churn–both reductions in the loss of insurance and lower movement between insurance–we see increased use of higher efficacy contraception postpartum, highlighting the critical role of Medicaid and insurance policies in providing insurance that can enable this contraceptive access across racial and ethnic groups and minimize insurance disruptions.
